# Cell-Autonomous Processes That Impair Xenograft Survival into the Cerebellum

**DOI:** 10.1007/s12311-022-01414-3

**Published:** 2022-05-16

**Authors:** Lorenzo Magrassi, Giulia Nato, Domenico Delia, Annalisa Buffo

**Affiliations:** 1 Neurosurgery, Department of Clinical, Surgical, Diagnostic and Pediatric Science, University of Pavia, Foundation IRCCS Policlinico San Matteo, Pavia, Italy; 2 Istituto Di Genetica Molecolare IGM-CNR, via Abbiategrasso 207, 27100 Pavia, Italy; 3 Neuroscience Institute Cavalieri Ottolenghi (NICO), Orbassano, 10043 Torino, Italy; 4 Department of Life Sciences and System Biology, University of Turin, Via Accademia Albertina 13, Turin, Italy; 5 IFOM, FIRC Institute of Molecular Oncology, Via Adamello 16, 20139 Milano, Italy; 6 Department of Neuroscience Rita Levi-Montalcini, University of Turin, Via Cherasco 15, Torino, Italy

**Keywords:** Xenotransplantation, Immune-tolerance, Cerebellum, Induced pluripotent stem cells

## Abstract

In immunocompetent animals, numerous factors including the immune system of the host regulate the survival of neuro-glial precursors transplanted into the cerebellum. We transplanted human neuro-glial precursors derived in vitro from partial differentiation of IPS cells into the developing cerebellum of mice and rats before maturation of the host immune system. These approaches should facilitate the development of immune-tolerance for the transplanted cells. However, we found that human cells survived the engraftment and integrated into the host cerebellum and brain stem up to about 1 month postnatally when they were rejected in both species. On the contrary, when we transplanted the same cells in NOD-SCID mice, they survived indefinitely. Our findings are consistent with the hypothesis that the slower pace of differentiation of human neural precursors compared to that of rodents restricts the induction of immune-tolerance to human antigens expressed before completion of the maturation of the immune system. As predicted by our hypothesis, when we engrafted the human neuro-glial precursor cells either in a more mature state or mixed with extracts from adult cerebellum, we prolonged the survival of the graft.

## The Xenotransplantation Approach to the Cerebellum

The development of reproducible techniques to generate induced pluripotent stem cells obtained from mature somatic cells of patients with specific neurologic conditions permits the study in vitro and in vivo of neural and glial precursors at different degrees of differentiation up to their full maturity [1, 2].

Despite the increasing use of organoids mimicking different areas of the brain and cerebellum [3, 4], orthotopically xenotransplanting human induced pluripotent stem cell-derived neural precursors (hiPSdNP) is still the only method to generate partially chimeric CNS where the xenografted human cells may integrate into the normal circuits of the host. This in vivo experimental model complements and extends the data that can be obtained by the same cells in vitro [5]. Furthermore, orthotopic xenotransplantation of human neural precursors into experimental animals represents an important preclinical step to test the translational potential of all neurotransplantation approaches developed to treat the diseases of the brain and cerebellum [6]. Unfortunately, one severe limitation to the xenotransplantation approach is the immune-reaction induced in the immunocompetent host by the xenotransplanted cells.

## Modulating the Immune-Reaction to the Xenografted Neural Precursors

The barrier that the host immune system raises is commonly overcome by transplanting the hiPSdNP into immune-depressed hosts [7] or by chronically treating immunocompetent animals with cyclosporine A [8] or tacrolimus [9]. However, both strategies have severe limitations when an extended survival of the graft is required. In fact, immunosuppressant drugs (e.g., cyclosporine A) require daily intraperitoneal injections and may damage the health of the host and—usually within 12–20 weeks of continuous treatment—they damage the health of the host so severely that the animal must be sacrificed [10]. Immune-depressed mice have also limitations when an extended survival of the human xenograft is important since their maximum lifespan is shorter than that of immunocompetent animals because they are prone to develop infections and neoplasms more often than their immunocompetent counterparts [11]. Moreover, there is a growing awareness that xenografting human cells or tissues into deeply immune-depressed mice (e.g., SCID mice) may not be optimal to model the presumptive behavior of the same cells when transplanted into homologous hosts since in a very short time (days) the host animal virome will substitute for the human one typical of the cells before transplantation [12]. Substitution of the donor virome with that of the host may have profound effects on the expression levels of many differentiation-, immune-, and drug metabolism-related genes in the transplanted cells [12].

## Inducing Immune-Tolerance

An alternative that promotes survival and differentiation of xenogenic neural precursors is based on the seminal observation of Billingham et al. [13] showing that active tolerance of xenotransplanted cells can be induced by engrafting cells of the same species of the donor before the complete development of the immune system. In practice, for common experimental rodents, the hosts of the grafts must be exposed to the cell of the donor during the fetal life or after birth up to the fifth postnatal day [13]. We have previously demonstrated the success of this experimental approach by orthotopic xenotransplantation of mouse cerebellar precursors into the developing rat cerebellum. In this experimental paradigm, the engrafted cerebellar neural precursors gave rise to neural (e.g., granule and Purkinje cells) and glial cells with typical cerebellar morphologies that were able to integrate into the host cerebellum and survive for the entire life of the host without immunosuppression [14]. An interesting variant to this approach of inducing immune-tolerance of xenografted neural precursors contemplates a double transplant of cells derived from the same species of the donor. The initial xenograft is used to induce immune-tolerance to the cells derived from the donor species and is performed heterotopically, usually intraperitoneally, before complete development of the host immune system; then, and after at least 8 weeks, the orthotopic intracerebral graft is performed [15]. In this experimental paradigm, the survival of the cells initially injected for tolerance induction is not necessary [16] since they are used only to induce immune-tolerance for the neural precursors injected after 8 weeks into the adult brain. However, this approach did not always result in long-term xenograft survival since rejection of the neural precursors orthotopically injected in the brain has been described according to the species and the strain of the host [16], the origin of the cells [17–19], and even the region in the brain targeted by the xenograft [18, 20].

## Immune-Tolerance to Xenografted Human Neural Precursors Is only Transient

**Fig. 1:**
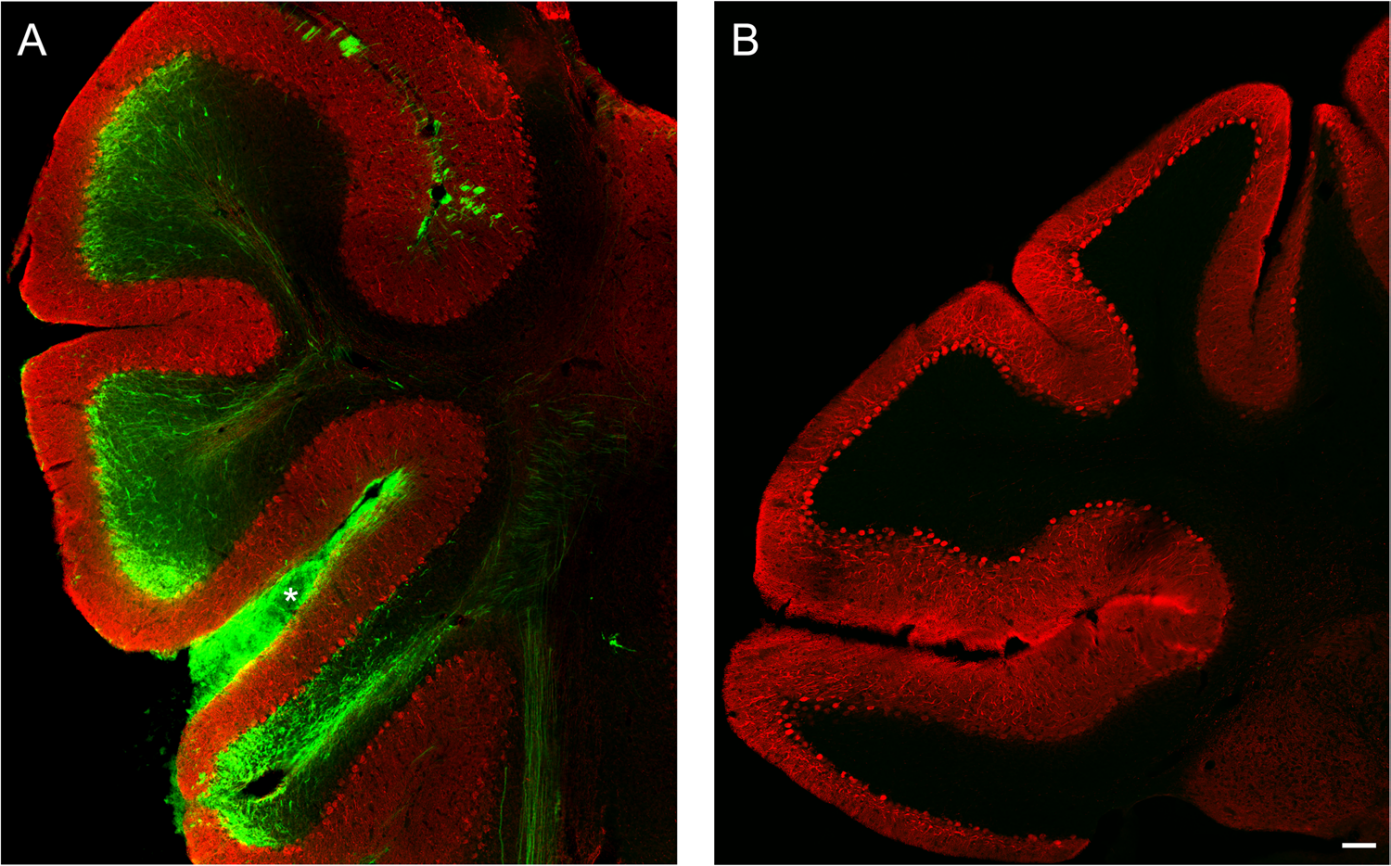
Mouse cerebellum sagittal sections. **A** Immune-deficient mouse (NOD-SCID: NOD.CB17-Prkdcscid/NCrHsd; Envigo) cerebellum 3 months after the transplant of hiPSdNP. Many processes and cells of human origin expressing green fluorescent protein are visible in the host cerebellar tissue and on the surface of the cerebellum (white asterisk), calbindine immunoreactivity is shown in red. **B** Immune-competent mouse (CD1) cerebellum 12 months after the transplant in utero of hiPSdNP, no cells of human origin expressing green fluorescent protein are visible, calbindine immunoreactivity is shown in red. We performed the experiments as described in Nato et al. [21]. Scale bar 100 µm

Recently, we tried to replicate our successful xenotransplantation and long-term survival of mouse cerebellar precursors in rat cerebellum after intra-utero transplantation [14] with human cerebellar precursors derived from in vitro differentiation of human induced pluripotent cells. However, we were unable to obtain survival of the donor cells in the rodent cerebellum longer than thirty postnatal days [21]. After destruction of the grafted human neural precursors, the cerebellum of the host was devoid of immune infiltration and microglia activation. Similar pathological findings have been observed in some cases of autoimmune-mediated cerebellar degeneration in humans when the elimination of Purkinje cells was complete [22]. However, the same cells transplanted into the cerebellar vermis of severely immune-depressed adult NOD-SCID mice survived and differentiated into neurons and glia well beyond 1 month after transplantation [21]. We also found that over time, human glial cells derived from the grafted hiPSdNP migrated throughout the whole cerebellum outcompeting the resident host glial cells. A similar behavior was demonstrated for human glial progenitors engrafted into the brain hemispheres of immune-depressed rodent hosts [23, 24]. This suggests that the engrafted hiPSdNP were able to survive, migrate, and differentiate after transplantation into the cerebellum of mice when protected from immune-rejection by the intrinsic immunodepression of the host (Fig. 1A). On the contrary, hiPSdNP xenografted into the developing cerebellum of immune-competent mice or rats survive and differentiate only up to about 1 month after birth when they are rejected (Fig. 1B).

But, why does xenografting in utero cerebellar precursors from mice into the rat cerebellum induces complete and lifelong immune-tolerance [14] while the very same experiment however, using human cerebellar precursors with survival and differentiation potentials similar to those of mice, results in complete immune-rejection of the transplant [21]?

## Difference in Developmental Times May Explain the Late Failure of Immune-Tolerance

These apparently contradictory results may be explained by considering that xenografted cerebellar precursors from both species differentiate in the host brain, following their species-specific developmental timing [21, 25]. Our findings are consistent with the hypothesis that the slower pace of differentiation of human neural precursors, compared to that of rodents, restricts the induction of immune-tolerance to human antigens expressed before completion of the maturation of the immune system. Conversely, more mature antigens that start to be expressed after the maturation of the host immune system are not recognized as self and therefore are rejected [21]. Due to the multiple developmental trajectories of the neuronal and glial populations present in the cerebellum, it is difficult to estimate the differential developmental time of the entire cerebellum across different mammalian species such as humans and mice. A global model integrating over one thousand neurodevelopmental events including some cerebellum-specific events such as onset and offset of Purkinje cell generation, peak generation of deep cerebellar nuclei, and superior cerebellar peduncle myelination onset [26] enables translating mouse and rat neurodevelopmental times into human ones. Focusing on a relatively well-known cerebellar developmental pathways such as Purkinje cell differentiation and dendritogenesis, a six–eightfold difference results between the time when Purkinje cells in humans demonstrate a well-developed and oriented dendrite with complete disappearance of somatic spines [27] and those same developmental milestones in mice [28, 29]. Interestingly, due to the more than one order of magnitude difference in the length of pregnancy between humans and mice, the development of Purkinje cell dendrites in mice is complete just before the end of the first postnatal month. However, in humans the same process is completed within the eighth month of fetal life [27]. This explains why—when we consider the absolute body length typical of the species, rather than the elapsed time—the milestones of the early cerebellar development in humans are reached at absolute body lengths which are intermediate between those of the mouse and the rat [30]. In our experiments, immune-rejection of the hiPSdNP xenografted in utero into the developing cerebellum took place around 1 month after birth of the host animal, when the host cerebellum has just reached its maturity. On the contrary, human cerebellar development is completed at the end of the first postnatal year. In our experimental paradigm, when we xenografted the same hiPSdNP mixed together with extracts from mature rat cerebellum, we prolonged the survival of the graft from 1 to 3 months [21]. This increase in survival time of the graft is significant if compared to that obtained by engrafting hiPSdNP alone. However, it is still shorter than that we obtained by xenografting the same cells in NOD-SCID mice [21]. Overall, these results indicate that developmental timing is a cell-autonomous process related to the species of origin of the xenografted cells.

## Immune Attack to Human Antigens Expressed by the Xenotransplanted Cells

Autoimmune attack to both mature and fetal cerebellar antigens has been described in several autoimmune diseases that affect humans, such as autism spectrum disorder [31, 32] and immune-mediated cerebellar ataxias [33]. Our experimental findings indicating late loss of tolerance to human cells engrafted in the developing cerebellum of rodents may be of interest to model some of the steps and mechanisms that lead to the autoimmune attacks on cerebellar cells both during development and in adulthood. Unfortunately, we still do not have suggestive indications of the human specific antigens involved in the delayed immune-rejection of the graft by the rodent hosts. However, it is interesting that, independently of the etiologies, the vast majority of the antigens identified as culprits in most cases of immune-mediated cerebellar ataxias are expressed in the mature cerebellum and not during cerebellar development [34].

## Concluding Remarks

There is an ongoing interest in modeling human neurological diseases by xenografting hiPSdNP into animal hosts [2, 5]. Our results point out that the ability to control the host immune system’s response is paramount to the long-term success and vitality of the xenografted cells and their descendants [21]. If we want to achieve the goal of prolonged survival of xenografted human cerebellar precursors into immune-competent hosts, then we must take into account the evolving nature of the transplant and the host immune system and modulate them accordingly.
